# Novel hypnotics use and hip fracture risk in middle‐aged and older adults: A large, population‐based cohort study in Japan

**DOI:** 10.1002/pcn5.70193

**Published:** 2025-08-18

**Authors:** Nana Shibata, Kazuhisa Yoshizawa, Masahiro Takeshima, Shingo Kitamura, Masaya Ogasawara, Mizuki Kudo, Yu Itoh, Eru Miyakoshi, Naoko Ayabe, Kazuo Mishima

**Affiliations:** ^1^ Department of Neuropsychiatry Akita University Graduate School of Medicine Akita City Akita Japan; ^2^ Department of Sleep‐Wake Disorders, National Center of Neurology and Psychiatry National Institute of Mental Health Kodaira Tokyo Japan; ^3^ Department of Regional Studies and Humanities, Faculty of Education and Human Studies Akita University Akita City Akita Japan

**Keywords:** benzodiazepines, hip fractures, hypnotic, melatonin, orexin

## Abstract

**Aim:**

To investigate the relationship between the prescription of novel hypnotics (melatonin receptor agonists [MRAs] and orexin receptor antagonists [ORAs]) and the risk of hip fractures in a large cohort of Japanese patients.

**Methods:**

In this retrospective study, we analyzed data from a large health insurance claims database. Among subscribers aged ≥50 years between April 2014 and September 2021, those assigned the disease code of hip fracture were included. Each patient's prescription history for hypnotics was examined to identify the exposure and non‐exposure periods. The relationship between exposure to hypnotics and the development of hip fractures was analyzed using the Mayo‐updated Cox proportional hazards regression model.

**Results:**

In total, 269,097 patients developed hip fractures. The prescription of any hypnotic was significantly associated with hip fracture incidence (adjusted hazard ratio [aHR], 2.30; 95% confidence interval [CI], 2.27–2.32). In the analysis by class of hypnotics, the hazard ratio was highest for ORAs (aHR, 3.09; 95% CI, 3.03–3.16), followed by that for MRAs (aHR, 2.45; 95% CI, 2.38–2.52).

**Conclusion:**

Novel hypnotics use was significantly associated with the development of hip fractures, and patients prescribed ORAs and MRAs should be cautioned about the risk of hip fractures.

## INTRODUCTION

Hip fracture is a serious condition that can lead to impaired daily functioning,^1^ reduced quality of life,^1^ and increased caregiver burden^2^ and societal costs.^3^ The incidence of hip fracture increases with age. As per a Japanese survey, the annual incidence rates per 10,000 hip fractures for men and women were 0.29 and 0.14 in those aged <40 years; 5.03 and 8.66 in those aged 60–69 years; and 159.5 and 323.3 in those aged >90 years, respectively.^4^ An increase has been observed in the percentage of the world's population aged ≥65 years (the aging rate) from 5.1% in 1950 to 9.4% in 2020. It is expected to increase to 18.7% in 2060, indicating that aging will accelerate rapidly over the next 40 years.^5^ As the number of patients with hip fractures is expected to increase in the future, identifying the risk factors for hip fractures and providing appropriate interventions are critical.

Hypnotic medication use is a major risk factor associated with hip fractures. A previous systematic review and meta‐analysis reported that benzodiazepines (BZs) and non‐benzodiazepines (NBZs), which are widely used worldwide, increased the risk of hip fracture by 1.52 and 1.9 times, respectively.^6^ Other drug‐specific and large‐scale analyses have reported significant relationships between BZs and NBZs and hip fracture.^7^, ^8^ In addition to fractures, the Beers Criteria established by the American Geriatrics Society lists BZs and NBZs as medications that may be inappropriate for older people because their use is associated with risks of cognitive dysfunction and delirium.^9^ Recently, novel hypnotics, such as orexin receptor antagonists (ORAs) and melatonin receptor agonists (MRAs), have been introduced. These novel hypnotics are safe because they are not addictive and have few side effects,^10^, ^11^, ^12^ and their effectiveness in preventing delirium has been reported.^13^, ^14^ However, the association between the risk of hip fractures and the use of novel hypnotics has not been fully elucidated, and the results of previous studies have been inconsistent.^15^, ^16^ A case–control study using reimbursement data from Japan's Medical Data Vision reported that suvorexant use was not associated with an increased risk of hip fracture.^15^ In contrast, a population‐based cohort study using reimbursement data from the Shizuoka Kokuho Database in Japan reported that suvorexant use was associated with a higher risk of hip fractures than the risk of BZ receptor agonist use (BZ and NBZ).^16^ However, the cohorts in these studies were small, and it is unclear whether they reflect the real‐world situation. As older people are prescribed hypnotics more often than younger people are,^17^ these prescriptions are increasing each year.^18^ The long‐term prescriptions of novel hypnotics are common^19^; therefore, an urgent need exists to investigate the risk of hip fractures with these medications. The results of previous studies must be validated using larger samples to determine whether the use of novel hypnotics increases the risk of hip fractures.

We conducted a retrospective cohort study using claims data from the DeSC database to examine the association between the risk of hip fractures and sleep medications, including novel sleep medications, in the older population.

## METHODS

### Study design and data sources

We performed a retrospective cohort study using the claims database provided by DeSC Healthcare Inc. (Tokyo, Japan). Information on age, sex, and the name of each prescribed psychotropic drug was collected for patients aged ≥50 years who were included in the database between April 2014 and September 2021.

The DeSC database contains claim data submitted to medical insurers in clinics, hospitals, and pharmacies between April 2014 and September 2021. The claims data included the patients' personal identification information, date of birth, sex, medical diagnosis according to the International Classification of Diseases Tenth Revision (ICD‐10) codes, prescribed medications, medical procedures, and surgery. The database comprised claims from three insurers: the Association Health Insurance (Kyokai Kenpo) for salaried workers in large companies; the National Health Insurance (Kokumin Kenko Hoken) for self‐employed individuals, retired workers, and their dependents; and the Late‐Stage Senior Citizen Medical Care System (Koki Koreisha Iryo Seido) for all persons aged ≥75. The population composition in this database is similar to that of the entire population of Japan and can be used for representative medical epidemiology.^20^

### Study population, outcomes, variables, and exposure

The study population consisted of insured individuals aged ≥50 years at the start of observation whose prescription status could be tracked. Patients in this population who sustained a hip fracture during the 90 months from April 2014 to September 2021 were selected. The prescription status of the psychotropic medications listed in Supporting Information S2: Table 1 was investigated for the selected patients. Patients were followed up until the first hip fracture, withdrawal from the insurer, death, or the end of the study period. The outcome was defined as hip fracture injury, defined by the first appearance of the ICD‐10 code S72 in the monthly medical data. The covariates included age, sex, and prescription of psychotropic medication (hypnotics, anxiolytics, antidepressants, antipsychotics, antiepileptics, antihistamines, or antidementia drugs). The exposure period was defined as the number of months hypnotics were prescribed during the analysis, whereas the other months were defined as the non‐exposure period. However, considering the duration of the effects of hypnotics, the months following the prescription month were considered the exposure period, even if hypnotics were not prescribed for that period.

### Statistical analysis

Continuous variables are presented as means (standard deviations), and nominal variables are presented as numbers (%). The Cox proportional hazards model was used to analyze hip fracture risk with hypnotics. The covariates used in proportional hazard models are usually limited to fixed factors and can be defined at the time of entry. However, these models are unsuitable while dealing with covariates that vary over time, such as those in this study. Accordingly, we used the Mayo‐updated model, a time‐dependent Cox proportional hazards model that can account for temporal changes in both exposure and covariates. In this model, multiple observation vectors were generated from a single patient and treated as independent intervals. This approach allowed for risk estimation by comparing hypnotics exposure and non‐exposure periods within the same individual at each time point. By design, this study is similar to the self‐controlled case series method and is considered to effectively control for time‐invariant unmeasured confounding.^21^ As a sensitivity analysis, we also restricted the analysis to individuals who received continuous hypnotic prescriptions for ≥6 months consecutively, assuming that this subgroup would reflect patients with more persistent or severe insomnia. The same time‐dependent Cox proportional hazards model was applied to this subgroup.

We used the proportional hazards model in the analysis by first performing an unadjusted risk analysis if any hypnotic was prescribed to outline the overall risk of hypnotics. An analysis adjusted for these covariates was performed to examine the effects of sex, age, and concomitant use of other psychotropics on hip fractures. Covariates for the multivariable model were selected based on clinical relevance and previous studies. Finally, unadjusted and adjusted analyses were performed for each class of hypnotic. Exposure to hypnotics was defined as both the month of prescription and the subsequent month, reflecting the pharmacologic duration of effect and the 30‐day dispensing rule in Japan. A simplified flowchart was added to clarify the time‐splitting and exposure classification process (Supporting Information S1: Figure 1). Analyses were performed using the Statistical Package for the Social Sciences (spss, version 28, Armonk, NY, USA), employing standard syntax for time‐to‐event modeling. The incidence of hip fractures is presented as a value per 10,000 person‐years. All the risk levels were set to 5%.

### Ethical considerations

The Ethics Committee of the Akita University Graduate School of Medicine and the Faculty of Medicine Ethical Committee for Human Research of our institution (No. 3086) approved this study. It was conducted according to the Declaration of Helsinki. As anonymized data were analyzed, the requirement for informed consent was waived.

## RESULTS

The study population comprised 6,851,882 patients, with a high proportion of women (Table 1). The mean age was 71.8 ± 10.5 years, with the largest proportion of patients in their 70s (32.8%). In this population, 269,097 patients had a hip fracture. The rate of hip fracture was higher in women than in men. The incidence of hip fractures increases with age.

**Table 1:** Characteristics of the cohort: the entire cohort and patients with hip fracture.

	All	Patients with hip fracture	Patients without hip fracture
	*N* = 6,851,882	*N* = 269,097	*N* = 6,582,785
*Sex*			
Female	3,838,103 (72.6%)	206,654 (82.6%)	3,631,449 (55.17%)
*Age*	71.8 (10.5)	81.76 (8.8)	71.4 (10.4)
*Age groups*			
50–59 years	891,645 (13.01%)	5049 (1.9%)	886,596 (13.47%)
60–69 years	2,103,801 (30.7%)	23,779 (8.8%)	2,080,022 (31.6%)
70–79 years	2,246,412 (32.79%)	65,467 (24.3%)	2,180,945 (33.13%)
80–89 years	1,319,395 (19.26%)	125,854 (46.8%)	1,193,541 (18.13%)
90–99 years	281,668 (4.11%)	47,416 (17.6%)	234,252 (3.56%)
≥100 years	8961 (0.13%)	1532 (0.6%)	7429 (0.11%)

*Note*: Values are presented as mean (standard deviation) or number (%).

The incidence of hip fractures per 10,000 person‐years was 123.88 (269,097 cases/21,721,684.08 person‐years) (Table 2). The incidence markedly increased with age and was similar in both sexes. The incidence was higher among women in all age groups.

**Table 2:** Incidence rate of hip fracture.

	All			Male			Female		
	HF	Person‐years	Incidence rate^a^	HF	Person‐years	Incidence rate^a^	HF	Person‐years	Incidence rate^a^
*All ages*	269,097	21,721,684.08	123.88	62,443	9,597,957.17	65.06	206,654	12,123,726.90	170.45
*Age groups*									
50–59 years	5049	2,997,685.00	16.84	2288	1,457,073.67	15.70	2761	1,540,611.33	17.92
60–69 years	23,779	8,145,907.00	29.19	8382	3,764,377.75	22.27	15,397	4,381,529.25	35.14
70–79 years	65,467	6,086,741.50	107.56	18,305	2,745,118.67	66.68	47,162	3,341,622.83	141.14
80–89 years	125,854	3,869,263.25	325.27	27,040	1,483,224.83	182.31	98,814	2,386,038.42	414.13
90–99 years	47,416	608,692.33	778.98	6318	146,544.17	431.13	41,098	462,148.17	889.28
≥100 years	1532	13,395.00	1143.71	110	1618.08	679.82	1422	11,776.92	1207.45

*Note*: Values are presented as numbers of HF.

Abbreviation: HF, hip fracture.

^a^ 10,000 person‐years.

Among the 269,097 patients with hip fractures, 152,443 (56.7%) were prescribed hypnotics (Supporting Information S2: Table 2). BZ was the most prescribed hypnotic, followed by NBZ, ORA, and MRA (34.1%, 29.9%, 19.30%, and 10.34%, respectively). The proportion of patients prescribed each class of hypnotic did not differ between male and female patients. Among psychotropic drugs other than hypnotics, antihistamines showed the highest percentage of prescriptions, followed by anxiolytics, antidementia, antipsychotics, and antidepressants. In contrast, antiepileptic drugs had the lowest percentage of prescriptions (40.36%, 22.1%, 21.9%, 21.4%, 19.3%, and 10.8%, respectively).

In 269,097 patients with hip fracture, the duration of exposure and non‐exposure to hypnotics was determined, and hip fracture risk was analyzed using the Mayo‐updated Cox proportional hazards regression model. Table 3 shows the hip fracture risk associated with hypnotics use, sex, age, and the concomitant use of other psychotropics in patients receiving hypnotics. Crude hazard ratios were derived from univariable Cox proportional hazards models. In the crude analysis, the hazard ratio associated with hypnotics was 2.23 (95% confidence interval [CI] 2.21–2.26). After adjusting for covariates, the hazard ratio associated with hypnotics was 2.30 (95% CI 2.27–2.32). Among concomitant drugs, the hazard ratio was highest with antipsychotics (1.30, 95% CI 1.28–1.32), followed by antiepileptics (1.14, 95% CI 1.12–1.17), antidementia drugs (1.10, 95% CI 1.08–1.12), and antidepressants (1.07, 95% CI 1.05–1.09). The concomitant use of anxiolytics (0.74, 95% CI 0.73–0.75) and antihistamines (0.98, 95% CI 0.96–0.99) was associated with a lower risk of hip fracture.

**Table 3:** Hip fracture risk in patients on hypnotics.

		Crude HR (95% CI)	Adjusted HR^a^ (95% CI)
Hypnotics		2.23 (2.21–2.26)*	2.30 (2.27–2.32)*
Sex	Male	Reference	Reference
	Female	1.06 (1.05–1.07)*	0.99 (0.98–1.00)
Age group	50–59 years	Reference	Reference
	60–69 years	1.01 (0.96–1.06)	1.09 (1.04–1.15)*
	70–79 years	1.21 (1.16–1.27)*	1.28 (1.22–1.34)*
	80–89 years	1.30 (1.25–1.36)*	1.35 (1.29–1.41)*
	90–99 years	1.76 (1.69–1.84)*	1.82 (1.74–1.91)*
	≥100 years	2.78 (2.55–3.03)*	2.74 (2.51–2.98)*
Anxiolytics		1.03 (1.02–1.05)*	0.74 (0.73–0.75)*
Antidepressants		1.34 (1.32–1.37)*	1.07 (1.05–1.09)*
Antipsychotics		1.61 (1.59–1.64)*	1.30 (1.28–1.32)*
Antiepileptics		1.30 (1.27–1.33)*	1.14 (1.12–1.17)*
Antihistamines		1.05 (1.03–1.07)*	0.97 (0.96–0.99)*
Antidementia drugs		1.27 (1.25–1.28)*	1.10 (1.08–1.12)*

*Note*: Asterisks indicate *p*‐values with significant results (*p* < 0.001).

Abbreviations: CI, confidence interval; HR, hazard ratio.

^a^ Adjustment: the Mayo‐updated Cox proportional hazards regression model was applied with the covariates of age, sex, hypnotics, anxiolytics, antidepressants, antipsychotics, antiepileptics, antihistamines, and antidementia drugs.

Table 4 shows the results of the analysis by class of hypnotic drug: hazard ratio for BZ was 1.52 (95% CI 1.50–1.54); NBZ, 2.01 (95% CI 1.98–2.03); MRA, 2.64 (95% CI 2.57–2.71); and ORA, 3.31 (95% CI 3.25–3.38). After adjusting for covariates, the hazard ratio for BZ was 1.59 (95% CI 1.57–1.62); NBZ, 1.96 (95% CI 1.94–1.99); MRA, 2.45 (95% CI 2.38–2.52); and ORA, 3.09 (95% CI 3.03–3.16).

**Table 4:** Hip fracture risk associated with prescriptions of each psychotropic agent.

	Crude HR (95% CI)	Adjusted^a^ HR (95% CI)
BZ	1.52 (1.50–1.54)*	1.59 (1.57–1.62)*
NBZ	2.01 (1.98–2.03)*	1.96 (1.94–1.99)*
MRA	2.64 (2.57–2.71)*	2.45 (2.38–2.52)*
ORA	3.31 (3.25–3.38)*	3.09 (3.03–3.16)*

*Note*: Asterisks indicate *p*‐values with significant results (*p* < 0.001).

Abbreviations: BZ, benzodiazepine; CI, confidence interval; HR, hazard ratio; MRA, melatonin receptor agonist; NBZ, non‐benzodiazepine; ORA, orexin receptor antagonist.

^a^ Adjustment: the Mayo‐updated Cox proportional hazards regression model was applied using the covariates of age, sex, hypnotics, anxiolytics, antidepressants, antipsychotics, antiepileptics, antihistamines, and antidementia drugs other than the drugs in the analysis.

As a sensitivity analysis, we restricted the population to patients who continuously received hypnotics prescriptions for at least 6 months (Supporting Information S2: Table 3). The association between hypnotics use and hip fracture risk remained consistent in direction and magnitude (adjusted hazard ratio 1.99, 95% CI 1.95–2.03). The association with certain covariates, such as antihistamine use, was attenuated and became statistically nonsignificant. Additionally, a class‐specific sensitivity analysis was conducted in the same cohort (Supporting Information S2: Table 4). The adjusted hazard ratios for BZ were 1.23 (95% CI 1.20–1.28); NBZ, 1.54 (95% CI 1.50–1.58); MRA, 1.92 (95% CI 1.83–2.01); and ORA, 2.35 (95% CI 2.26–2.44). These estimates were comparable to those observed in the main analysis.

## DISCUSSION

This study is the first to investigate the relationship between hypnotics, including novel hypnotics, and hip fracture risk in a single large cohort. The population and sex composition of this cohort are comparable to those of Japan.^20^ These results can be considered reliable real‐world data for Japan.

The Mayo‐updated Cox proportional hazards regression model showed a significantly higher risk of hip fracture during periods when hypnotics were prescribed than during periods when no hypnotics were prescribed. In the analysis by hypnotic class, ORAs had the highest hazard ratio of 3.09, followed by MRAs at 2.45. The exact reason why the hazard ratio was higher for novel hypnotics than that of conventional hypnotics is unclear. Novel hypnotics may cause less sedation than conventional hypnotics, which may increase the frequency of somnolent walking and increase the risks of falls and fractures. In addition, the risk of falls associated with BZ use has been well documented in recent years; as a result, patients whose physicians were willing to prescribe ORAs might have been at a greater risk of experiencing falls and developing fractures. A survey of Japanese physicians indicated that those who frequently prescribe ORAs place more emphasis on efficacy and safety when selecting a drug than those who do not prescribe ORAs.^22^ Comorbidities were not considered in this study, and further studies with additional confounding factors are warranted.

The results of previous studies on the novel hypnotic ORA have been inconsistent. One case–control study reported that ORA use was not associated with hip fracture risk. However, this study only included inpatients, suggesting that the control group also had more underlying diseases.^15^ Similar to the present study, a retrospective cohort study using an insurance claims database reported that the ORA‐treated group did not have an increased risk of hip fracture compared with that in the BZ‐treated group^23^ and that the risk was higher than that in the BZ‐ and NBZ‐treated groups.^16^ Although direct comparisons cannot be made in this study, as it compares the periods of drug administration and non‐administration in the same patients, hip fractures should be noted in ORA‐treated patients. This study also included the novel ORA lemborexant in the analysis so that the results may be more reflective of the real‐world situation. Few studies have been performed on ramelteon, a novel hypnotic classified as an MRA. Melatonin was associated with a higher risk of hip fracture,^24^ and only patients who were prescribed melatonin three or more times had a higher risk of developing hip fractures than patients who were not prescribed hypnotics. The number of prescriptions was not investigated in this study, and the association of the number of prescriptions with long‐term continuous use should be investigated in the future. A recent report compared melatonin and BZ groups after propensity score matching and found no difference in hip fracture risk between the groups.^25^ The relationship between melatonin and fractures is not limited to falls. A mechanism by which the activation of melatonin Receptor 2 improves osteoporosis by promoting bone formation and inhibiting bone resorption is known.^26^ Although ramelteon itself has a relatively short half‐life, typically cited as 1 h, it is metabolized into an active metabolite, M‐II, which has a significantly longer half‐life and greater systemic exposure. This may contribute to prolonged pharmacologic effects and possibly a higher risk of falls.^27^ Similarly, ORAs are also known to have relatively longer half‐lives, which may result in residual sedative effects. Further research is required to clarify whether these pharmacokinetic properties directly contribute to fall risk in real‐world settings.

As a sensitivity analysis, we restricted the population to patients who received hypnotics prescriptions continuously for at least 6 months. The association between hypnotics use and hip fracture risk remained statistically significant and consistent with the main findings, suggesting the robustness of the results. Furthermore, when the same sensitivity analysis was conducted for each class of hypnotics, a similar gradient of risk was observed. These findings support the consistency of risk differences across classes of hypnotics and suggest that a comparable risk exists even in more severe patients who are prescribed hypnotics over a longer period.

Among the combinations of hypnotics and other psychotropic medications, antipsychotics had the strongest association with the increased risk of hip fractures. The risk of hip fracture with antipsychotics has also been reported,^28^ and the risk may further increase with the concomitant use of hypnotics. Antipsychotic drugs are associated not only with sleepiness but also with an increased risk of falls because of orthostatic hypotension, as well as a decrease in bone density because of hyperprolactinemia.^29^ Antidepressants and antiepileptic drugs have also been associated with decreased bone density,^30^, ^31^ suggesting that the concomitant use of hypnotics with these drugs may further increase the fracture risk. The mechanism by which the concomitant use of anxiolytics and antihistamines is associated with a lower risk of hip fracture is unknown. However, these drugs can have an effect, as anxiolytics include tandospirone, a non‐sedating drug, and antihistamines include various drugs with varying degrees of sedation. Further drug‐specific studies are warranted.

This study had several limitations. First, because only the prescription of hypnotics was used as the exposure factor, the study failed to consider the fall and fracture risk of insomnia itself. For older adults with reduced activities of daily living, insomnia is associated with a risk of falling at night.^32^ A Danish self‐controlled case series reported that the risk of fractures was higher among patients with insomnia in the period just before initiation of treatment with hypnotics.^33^ Second, the comorbidities of patients were not considered. Several diseases affect falls and fractures, including medical diseases that affect general health, orthopedic diseases that affect unsteadiness, and eye diseases. Third, we cannot validate whether the patient was actually consuming the medication because the analysis was based on insurance claims data. As the exposure factors in this study were extracted based on whether the patient was prescribed the drug, the actual rate of oral medication remains unknown. Fourth, the outcome, the occurrence of hip fracture, was also extracted from the disease names in the insurance claims data. Even if a fracture had occurred, it would not have been included in this analysis if it had not been given a disease name in the insurance claims data; therefore, the possibility of underestimation cannot be overlooked. Fifth, the dose and duration of drug administration were not considered. The effects of many hypnotics vary in a dose‐dependent manner. In this analysis, we were unaware whether the drugs were administered at the lowest or highest dose. Additionally, we did not know the time of administration, whether the administration was in the evening or late at night. Therefore, further prospective studies are required. Sixth, although the time‐dependent Cox model allows for within‐individual comparisons and adjustment for time‐invariant confounders, residual confounding and prescription bias cannot be fully excluded. For example, clinicians may preferentially prescribe ORAs to patients with more severe insomnia, frailty, or psychiatric comorbidities, potentially contributing to the elevated hazard ratio observed for ORAs. To partially address this concern, we conducted a sensitivity analysis restricted to patients who received hypnotics prescriptions for ≥6 months continuously. The results were consistent with those of the main analysis, suggesting the robustness of our findings. However, as this approach does not account for all sources of bias, further research is warranted.

In conclusion, the analysis of a large Japanese database revealed a relationship between hypnotic prescriptions and the risk of hip fracture development. Novel hypnotics have also been suggested to be associated with the risk of developing hip fractures, and we should be aware of the risk of hip fractures associated with hypnotic prescriptions.

## PERMISSION TO REPRODUCE MATERIAL FROM OTHER SOURCES

There is no reproduced material from other sources in this article.

## AUTHOR CONTRIBUTIONS

All authors contributed substantially to the conception and design of the study and the acquisition, analysis, and interpretation of the data. All authors contributed to the drafting of the manuscript for important intellectual content. All authors agree to be accountable for all aspects of this study.

## CONFLICT OF INTERESTS STATEMENT

Nana Shibata has received speaker honoraria from Otsuka Pharmaceutical, outside the submitted work. Kazuhisa Yoshizawa has received speaker honoraria from Meiji Seika Pharma, EISAI Co., Ltd., Kowa Co., Ltd., Otsuka Pharmaceutical, and Janssen Pharmaceutical K.K., outside the submitted work. Masahiro Takeshima received speaker honoraria from Takeda Pharmaceutical Co., Ltd., Otsuka Pharmaceutical, Daiichi Sankyo Company, Sumitomo Pharma Co., Ltd., Meiji Seika Pharma, Viatris Pharmaceuticals Japan, MSD, EISAI Co., Ltd., Yoshitomi Pharmaceutical, and Janssen Pharmaceutical K.K., outside the submitted work. Shingo Kitamura received research grants from Mitsui Fudosan Co., Ltd. and Tsubota Laboratory Inc., outside the submitted work. Masaya Ogasawara received speaker honoraria from Sumitomo Pharma Co., Ltd., outside the submitted work. Mizuki Kudo received speaker honoraria from Meiji Seika Pharma Co., Ltd. and Otsuka Pharmaceutical, outside the submitted work. Yu Itoh received speaker honoraria from Sumitomo Pharma Co., Ltd., outside the submitted work. Naoko Ayabe received speaker honoraria from MSD and EISAI Co. Ltd., outside the submitted work. Kazuo Mishima received speaker honoraria from EISAI Co., Ltd., Nobel Pharma Co., Ltd., Takeda Pharmaceutical Co., Ltd., and MSD Inc., and research grants from EISAI Co., Ltd., Sumitomo Pharma Co., Ltd., and Takeda Pharmaceutical Co., Ltd. Eru Miyakoshi declares no conflicts of interest.

## ETHICS APPROVAL STATEMENT

This study was approved by the Ethics Committee of the Akita University Graduate School of Medicine and the Faculty of Medicine Ethical Committee for Human Research of our institution (no. 3086).

## PATIENT CONSENT STATEMENT

The need for patient consent was waived because of the retrospective study design and the use of anonymized DeSC data.

## CLINICAL TRIAL REGISTRATION

N/A.

## Supporting information

Supporting Information.

Supporting Information.

## Data Availability

The data supporting this study's findings are available from the DeSC, but restrictions apply to the availability of these data. The data were used under license for the current study and, thus, are not publicly available. The data are available from the authors upon reasonable request and with permission from the DeSC.
