# Supplementary figures and images for: Novel hypnotics use and hip fracture risk in middle‐aged and older adults: A large, population‐based cohort study in Japan
Source: PCN Rep. 2025 Aug 18;4(3):e70193. doi: 10.1002/pcn5.70193 (PMC12360458; doi:10.1002/pcn5.70193)

Figure S1

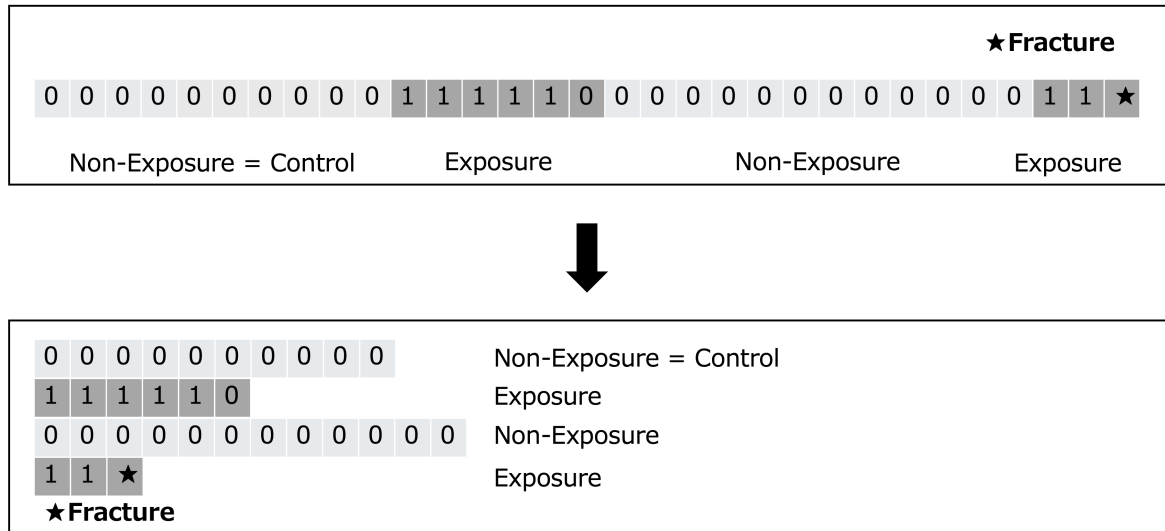

Supplement: Supplementary file 1 — Supporting Information. [file PCN5-4-e70193-s001.pdf]
