# Supplementary material for: Novel hypnotics use and hip fracture risk in middle‐aged and older adults: A large, population‐based cohort study in Japan
Source: PCN Rep. 2025 Aug 18;4(3):e70193. doi: 10.1002/pcn5.70193 (PMC12360458; doi:10.1002/pcn5.70193)
Supplement: Supplementary file 2 — Supporting Information. [file PCN5-4-e70193-s002.docx]

**Figure S1.** Monthly exposure classification based on hypnotics prescription timing in the Mayo-updated cox model

Schematic illustration of the time-splitting and exposure classification process in the Mayo-updated Cox proportional hazards model. Each participant’s follow-up period was divided into monthly intervals. Exposure to hypnotics was defined as both the month of prescription and the subsequent month, in accordance with the pharmacologic effect duration and the 30-day dispensing rule in Japan.
1 = month with hypnotics prescription; 0 = month without hypnotics prescription.

**Table S1.** List of psychotropics covered in this study

**Hypnotics**

| Generic name | ATC code | Class |
| --- | --- | --- |
| Brotizolam | N05CD09 | Benzodiazepines (BZs) |
| Estazolam | N05CD04 |  |
| Etizolam | N05BA19 |  |
| Flunitrazepam | N05CD03 |  |
| Flurazepam | N05CD01 |  |
| Haloxazolam | － |  |
| Lormetazepam | N05CD06 |  |
| Nimetazepam | N05CD15 |  |
| Nitrazepam | N05CD02 |  |
| Quazepam | N05CD10 |  |
| Rilmazafone | － |  |
| Triazolam | N05CD05 |  |
| Zolpidem | N05CF02 | Non-benzodiazepines (NBZs) |
| Zopiclone | N05CF01 |  |
| Eszopiclone | N05CF04 |  |
| Ramelteon | N05CH02 | Melatonin receptor agonist (MRA) |
| Suvorexant | N05CM19 | Orexin receptor antagonists (ORAs) |
| Lemborexant | N05CJ02 |  |
| Amobarbital | N05CA02 | Others |
| Barbital | N05CA04 |  |
| Bromovalerylurea | － |  |
| Butoctamide | － |  |
| Chloral hydrate | N05CC01 |  |
| Passiflora extract | － |  |
| Pentobarbital calcium | N05CA01 |  |
| Phenobarbital | N03AA02 |  |
| Secobarbital | N05CA06 |  |
| Triclofos sodium | N05CM07 |  |

ATC, the Anatomical Therapeutic Chemical classification 2021

**Anxiolytics**

| Generic name | ATC code |
| --- | --- |
| Alprazolam | N05BA12 |
| Bromazepam | N05BA08 |
| Chlordiazepoxide | N05BA02 |
| Clorazepate | N05BA05 |
| Clotiazepam | N05BA21 |
| Cloxazolam | N05BA22 |
| Diazepam | N05BA01 |
| Etizolam | N05BA19 |
| Fludiazepam | N05BA17 |
| Flutazolam | － |
| Flutoprazepam | － |
| Hydroxyzine | N05BB01 |
| Loflazepate | N05BA18 |
| Lorazepam | N05BA06 |
| Medazepam | N05BA03 |
| Mexazolam | N05BA25 |
| Oxazepam | N05BA04 |
| Oxazolam | － |
| Prazepam | N05BA11 |
| Tandospirone | － |
| Tofisopam | N05BA23 |

ATC, the Anatomical Therapeutic Chemical classification 2021

Antidepressants

| Generic name | ATC code |
| --- | --- |
| Amitriptyline | N06AA09 |
| Amoxapine | N06AA17 |
| Clomipramine | N06AA04 |
| Desipramine | N06AA01 |
| Dosulepin | N06AA16 |
| Duloxetine | N06AX21 |
| Escitalopram | N06AB10 |
| Fluvoxamine | N06AB08 |
| Imipramine | N06AA02 |
| Lofepramine | N06AA07 |
| Maprotiline | N06AA21 |
| Mianserin | N06AX03 |
| Milnacipran | N06AX17 |
| Mirtazapine | N06AX11 |
| Nortriptyline | N06AA10 |
| Paroxetine | N06AB05 |
| Safrazine | － |
| Sertraline | N06AB06 |
| Setiptiline | － |
| Sulpiride | N05AL01 |
| Trazodone | N06AX05 |
| Trimipramine | N06AA06 |
| Venlafaxine | N06AX16 |
| Vortioxetine | N06AX26 |

ATC, the Anatomical Therapeutic Chemical classification 2021

**Antipsychotics**

| Generic name | ATC code |
| --- | --- |
| Aripiprazole | N05AX12 |
| Asenapine | N05AH05 |
| Blonanserin | － |
| Brexpiprazole | N05AX16 |
| Bromperidol | N05AD06 |
| Carpipramine | － |
| Chlorpromazine | N05AA01 |
| Clocapramine | － |
| Clozapine | N05AH02 |
| Floropipamide | － |
| Fluphenazine | N05AB02 |
| Haloperidol | N05AD01 |
| Levomepromazine | N05AA02 |
| Lurasidone | N05AE05 |
| Moperone | N05AD04 |
| Mosapramine | N05AX10 |
| Nemonapride | － |
| Olanzapine | N05AH03 |
| Oxypertine | N05AE01 |
| Paliperidone | N05AX13 |
| Perospirone | － |
| Perphenazine | N05AB03 |
| Pimozide | N05AG02 |
| Prochlorperazine | N05AB04 |
| Propericyazine | － |
| Quetiapine | N05AH04 |
| Risperidone | N05AX08 |
| Spiperone | － |
| Sulpiride | N05AL01 |
| Sultopride | N05AL02 |
| Tiapride | N05AL03 |
| Thioridazine | N05AC02 |
| Timiperone | － |
| Trifluoperazine | N05AB06 |
| Zotepine | N05AX11 |

ATC, the Anatomical Therapeutic Chemical classification 2021

**Antiepileptics**

| Generic name | ATC code |
| --- | --- |
| Acetazolamide Sodium | S01EC01 |
| Acetylpheneturide | － |
| Carbamazepine | N03AF01 |
| Clobazam | N05BA09 |
| Clonazepam | N03AE01 |
| Ethosuximide | N03AD01 |
| Ethotoin | N03AB01 |
| Fosphenytoin sodium hydrate | N03AB05 |
| Gabapentin | N02BF01 |
| Lacosamide | N03AX18 |
| Lamotrigine | N03AX09 |
| Levetiracetam | N03AX14 |
| Metarbital | － |
| Oxcarbazepine | N03AF02 |
| Perampanel Hydrate | N03AX22 |
| Phenobarbital | N03AA02 |
| Phenytoin | N03AB02 |
| Piracetam | N06BX03 |
| Primidone | N03AA03 |
| Rufinamide | N03AF03 |
| Sodium Valproate | N03AG01 |
| Stiripentol | N03AX17 |
| Sultiame | N03AX03 |
| Topiramate | N03AX11 |
| Trimethadione | N03AC02 |
| Vigabatrin | N03AG04 |
| Zonisamide | N03AX15 |

ATC, the Anatomical Therapeutic Chemical classification 2021

**Antihistamines**

| Generic name | ATC code |
| --- | --- |
| Alimemazine | R06AD01 |
| Astemizole | R06AX11 |
| Azelastine | R06AX19 |
| Bepotastine | － |
| Bilastine | R06AX29 |
| Cetirizine | R06AE07 |
| Clemastine | R06AA04 |
| Cyproheptadine | R06AX02 |
| d-chlorpheniramine | R06AB02 |
| Desloratadine | R06AX27 |
| Dimenhydrinate | R06AA11 |
| Diphenhydramine | R06AA02 |
| Diphenylpyraline | R06AA07 |
| dl-chlorpheniramine | － |
| Ebastine | R06AX22 |
| Emedastine | S01GX06 |
| Epinastine | R06AX24 |
| Fexofenadine | R06AX26 |
| Homochlorcyclizine | － |
| Hydroxyzine Hydrochloride | － |
| Ketotifen | R06AX17 |
| Levocetirizine | R06AE09 |
| Levocetirizine/pseudoephedrine | － |
| Loratadine | R06AX13 |
| Mequitazine | R06AD07 |
| Olopatadine | R01AC08 |
| Oxatomide | R06AE06 |
| Promethazine Hydrochloride | R06AD02 |
| Rupatadine Fumarate | R06AX28 |
| Terfenadine | R06AX12 |
| Triprolidine | R06AX07 |

ATC, the Anatomical Therapeutic Chemical classification 2021

**Antidementia drugs**

| Generic name | ATC code |
| --- | --- |
| Donepezil hydrochloride | N06DA02 |
| Galantamine hydrobromide | N06DA04 |
| Memantine hydrochloride | N06DX01 |
| Rivastigmine | N06DS03 |

ATC, the Anatomical Therapeutic Chemical classification 2021

**Table S2.** Psychotropic prescriptions for patients with hip fracture (N=269,097)

|  | All |  | Male |  | Female |
| --- | --- | --- | --- | --- | --- |
|  | N=269,097 |  | N=62,443 |  | N=206,654 |
| Hypnotics | 152,443(56.7%) |  | 34,685(55.6%) |  | 117,758(57.0%) |
| BZ | 91,790(34.1%) |  | 20,344(32.6%) |  | 71,446(34.6%) |
| NBZ | 80,577(29.9%) |  | 18,328(29.4%) |  | 62,249(30.1%) |
| MRA | 27,820(10.34%) |  | 7,061(11.3%) |  | 20,759(10.1%) |
| ORA | 51,814(19.3%) |  | 12,757(20.4%) |  | 39,057(18.9%) |
| Anxiolytics | 59,405(22.1%) |  | 11,911(19.1%) |  | 47,494(23.0%) |
| Antidepressants | 51,986(19.3%) |  | 10,798(17.3%) |  | 41,188(19.9%) |
| Antipsychotics | 57,486(21.4%) |  | 13,635(21.8%) |  | 43,851(21.2%) |
| Antiepileptics | 28,953(10.8%) |  | 8,523(13.7%) |  | 20,430(9.9%) |
| Antihistamines | 108,599(40.36%) |  | 27,020(43.3%) |  | 81,579(39.5%) |
| Antidementia drugs | 58,948(21.9%) |  | 11,149(17.9%) |  | 47,799(23.1%) |

Note: Values are presented as numbers (%).

Abbreviations: BZ, benzodiazepine; MRA, melatonin receptor agonist; NBZ, non-benzodiazepine; ORA, orexin receptor antagonist

**Table S3.** Sensitivity analysis of hip fracture risk in patients with continuous hypnotic prescriptions for ≥6 months

|  |  | Crude HR (95% CI) |  | Adjusted HR^†^(95% CI) |
| --- | --- | --- | --- | --- |
| Hypnotics |  | 1.94(1.90–1.98)* |  | 1.99(1.95–2.03)* |
| ***Sex*** | Male | Reference |  | Reference |
|  | Female | 1.07(1.05–1.09)* |  | 1.03(1.01–1.05)* |
| ***Age group*** | 50–59y | reference |  | reference |
|  | 60–69y | 1.02(0.95–1.09) |  | 1.10(1.03–1.18)* |
|  | 70–79y | 1.19(1.12–1.27)* |  | 1.31(1.23–1.40)* |
|  | 80–89y | 1.25(1.17–1.33)* |  | 1.38(1.29–1.47)* |
|  | 90–99y | 1.68(1.57–1.79)* |  | 1.85(1.74–1.98)* |
|  | ≥100 | 2.49(2.17–2.86)* |  | 2.70(2.34–3.10)* |
| Anxiolytics |  | 0.92(0.91–0.94)* |  | 0.80(0.79–0.82)* |
| Antidepressants |  | 1.18(1.15–1.20)* |  | 1.07(1.04–1.09)* |
| Antipsychotics |  | 1.37(1.34–1.40)* |  | 1.26(1.23–1.29)* |
| Antiepileptics |  | 1.22(1.19–1.27)* |  | 1.17(1.13–1.20)* |
| Antihistamines |  | 1.03(1.01–1.06)* |  | 1.01(0.99–1.03) |
| Antidementia drugs |  | 1.24(1.21–1.27)* |  | 1.14(1.11–1.16)* |

^†^Adjustment: The Mayo-updated Cox proportional hazards regression model was applied with the covariates of age, sex, hypnotics, anxiolytics, antidepressants, antipsychotics, antiepileptics, antihistamines, and antidementia drugs.

Note: Asterisks indicate p-values with significant results (p < 0.001).

Abbreviations: CI, confidence interval; HR, hazard ratio

**Table S4.** Sensitivity analysis of hip fracture risk associated with prescriptions of each hypnotics class in patients with continuous hypnotic prescriptions for ≥6 months

|  | Crude HR (95% CI) |  | Adjusted^†^ HR (95% CI) |
| --- | --- | --- | --- |
| BZ | 1.17(1.14–1.20)* |  | 1.23(1.20–1.28)* |
| NBZ | 1.55(1.51–1.60)* |  | 1.54(1.50–1.58)* |
| MRA | 2.01(1.97–2.15)* |  | 1.92(1.83–2.01)* |
| ORA | 2.51(2.42–2.60)* |  | 2.35(2.26–2.44)* |

^†^Adjustment: The Mayo-updated Cox proportional hazards regression model was applied using the covariates of age, sex, hypnotics, anxiolytics, antidepressants, antipsychotics, antiepileptics, antihistamines, and antidementia drugs other than the drugs in the analysis.

Note: Asterisks indicate p-values with significant results (p < 0.001).

Abbreviations: BZ, benzodiazepine; CI, confidence interval; HR, hazard ratio; MRA, melatonin receptor agonist; NBZ, non-benzodiazepine; ORA, orexin receptor antagonist
